# Olfactory ensheathing cells in facial nerve regeneration^☆^

**DOI:** 10.1016/j.bjorl.2018.07.006

**Published:** 2018-08-07

**Authors:** Manyi Li, Qiubei Zhu, Jisheng Liu

**Affiliations:** a Soochow University, The First Affiliated Hospital, Department of Otorhinolaryngology, Suzhou, China; b Shanghai Changzheng Hospital, Department of Otorhinolaryngology Head and Neck Surgery, Shanghai, China

**Keywords:** Facial nerve, Olfactory bulb, Tissue engineering, Nervo facial, Bulbo olfatório, Engenharia de tecidos

## Abstract

**Introduction:**

Olfactory ensheathing cell is a unique kind of glia cells, which can promote axon growth. Little is known about the differences between olfactory mucosa olfactory ensheathing cells and olfactory bulb olfactory ensheathing cells in the capability to promote nerve regeneration.

**Objective:**

To study the recovery of the rat facial nerve after olfactory ensheathing cells transplantation, and to compare the differences between the facial nerve regeneration of olfactory mucosa-olfactory ensheathing cells and olfactory bulb olfactory bulb olfactory ensheathing cells transplantation.

**Methods:**

Institutional ethical guideline was followed (201510129A). Olfactory mucosa-olfactory ensheathing cells and olfactory bulb olfactory ensheathing cells were cultured and harvested after 7 days in vitro. 36 *Sprague Dawley* male rats were randomly divided into three different groups depending on the transplanting cells: Group A: olfactory mucosa-olfactory ensheathing cells; Group B: olfactory bulb olfactory ensheathing cells; Group C: DF-12 medium/fetal bovine serum. The main trunk of the facial nerve was transected and both stumps were inserted into a polylactic acid/chitosan conduit, then the transplanted cells were injected into the collagen in the conduits. After 4 and 8 weeks after the transplant, the rats of the three groups were scarified and the facial function score, facial nerve evoked potentials, histology analysis, and fluorescent retrograde tracing were tested and recorded, respectively, to evaluate the facial nerve regeneration and to analysis the differences among the three groups.

**Results:**

Olfactory ensheathing cells can promote the facial nerve regeneration. Compared with olfactory bulb olfactory ensheathing cells, olfactory mucosa olfactory ensheathing cells were more effective in promoting facial nerve regeneration, and this difference was more significant 8 weeks after the transplantation than 4 weeks.

**Conclusion:**

We discovered that olfactory ensheathing cells with nerve conduit could improve the facial nerve recovery, and the olfactory mucosa olfactory ensheathing cells are more effective for facial nerve regeneration compared with olfactory bulb olfactory ensheathing cells 8 weeks after the transplantation. These results could cast new light in the therapy of facial nerve defect, and furnish the foundation of auto-transplantation of olfactory mucosa olfactory ensheathing cells in periphery nerve injury.

## Resumo

**Introdução:**

A célula embainhante olfatória é um tipo especial de célula glial que pode promover o crescimento do axônio. Pouco se sabe sobre as diferenças entre as células embainhantes olfatórias da mucosa olfatória e as células embainhantes olfatórias do bulbo olfatório em relação à sua capacidade de promover a regeneração nervosa.

**Objetivo:**

Estudar a regeneração do nervo facial de ratos após o transplante de células embainhantes olfatórias e comparar as diferenças entre a regeneração do nervo facial com o transplante de células embainhantes olfatórias da mucosa olfatória e de células embainhantes olfatórias do bulbo olfatório.

**Método:**

As recomendações éticas da instituição (201510129A) foram seguidas. Células embainhantes olfatórias da mucosa olfatória e células embainhantes olfatórias do bulbo olfatório foram cultivadas *in vitro* e coletadas após sete dias. Trinta e seis ratos *Sprague Dawley* machos foram divididos aleatoriamente em três grupos, dependeu das células transplantadas: Grupo A, células embainhantes olfatórias da mucosa olfatória; Grupo B, células embainhantes olfatórias do bulbo olfatório; Grupo C, meio de DF-12/soro fetal bovino. O tronco principal do nervo facial foi seccionado e ambos os cotos foram inseridos em um conduto de ácido polilático/quitosana; em seguida, as células transplantadas foram injetadas em colágeno nos condutos. Após quatro e oito semanas do transplante, os ratos dos três grupos foram agitados para a obtenção do escore da função facial, potenciais evocados do nervo facial, análise histológica e marcador fluorescente retrógrado, que foram testados e registrados, respectivamente, para avaliar a regeneração do nervo facial e analisar as diferenças entre os três grupos.

**Resultados:**

Células embainhantes olfatórias podem promover a regeneração do nervo facial. Em comparação com as células embainhantes olfatórias do bulbo olfatório, as células embainhantes olfatórias da mucosa olfatória foram mais eficazes na promoção da regeneração do nervo facial, e essa diferença foi mais significativa oito semanas após o transplante em comparação com quatro semanas.

**Conclusão:**

Verificamos que células embainhantes olfatórias com conduto nervoso podem melhorar a recuperação do nervo facial e as células embainhantes olfatórias da mucosa olfatória são mais eficazes para a regeneração do nervo facial em comparação com as células embainhantes olfatórias do bulbo olfatório oito semanas após o transplante. Esses resultados podem lançar uma nova luz no tratamento de defeitos do nervo facial e fornecer a base do autotransplante de células embainhantes olfatórias da mucosa olfatória em lesões do nervo periférico.

## Introduction

Facial nerve defect can severely affect patients’ quality of life.^1^ Treatments include surgical reinnervation procedures (end-to-end anastomosis), autologous nerve grafts, etc. However, many treatments have severe side effects, and the facial motor function can hardly return to normal after surgery.^2^

Peripheral nerves do have the potential to regenerate after injury. Current strategies to repair injured peripheral nerve system focus on developing bridging scaffolds that could guide axonal regeneration across the lesion site. With the development of tissue engineering, it is possible to use nerve conduits, grafts filling material, and appropriate seed cells to bridge the injured peripheral nerve and to promote nerve regeneration.^2^ Previous studies showed that the outcome of peripheral nerve regeneration using tissue engineering is similar to that of autogenous nerve transplantation.^3^

Olfactory ensheathing cell (OEC) is a unique kind of glia cells, which can promote axon growth.^4^ OECs can maintain somatotopic organization after lesion, promote regeneration of olfactory nerve fibers, and improve the organization as well as the quantity and speed of nerve regeneration.^5^, ^6^, ^7^ Many studies showed that OECs transplantation has a promising therapeutic value in central and peripheral nerve injury, especially in spinal cord injury.^6^, ^8^

Finding a reliable source of OECs which is easily accessible and can ensure a sufficient number of cells is a major prerequisite for conducting studies on OEC-mediated nerve regeneration. Most previous studies obtained OECs from the Olfactory Bulb (OB) within the cranial cavity.^5^ OECs can also be isolated from the Olfactory Mucosa (OM),^9^, ^10^ which may provide a more readily available source of these cells for autologous transplantation. Previous studies showed that OECs obtained from the OM were therapeutic when transplanted into the injured Central Nerve System (CNS) or Peripheral Nerve System (PNS) of patients and animal models.^11^, ^12^, ^13^, ^14^, ^15^ In addition, there is no associated comorbidity since it has been shown that olfactory biopsies do not impair the sense of smell.^16^

Previous studies showed that OM-OECs and OB-OECs both have the capability to promote nerve regeneration, but little is known about their differences. In this study, we transplanted different originated OECs (OM-OECs and OB-OECs) in PLA/chitosan conduit to bridge the defected facial nerve in rat, and compared the differences between the function of inducing nerve regeneration of OM-OECs and OB-OECs. Behavioral, electrophysiology, histology, and retrograde tests were used to assess the outcomes.

## Methods

### OECs preparation

OECs were isolated, cultured, and purified from rat olfactory mucosa and olfactory bulb, respectively.

OM-OECs were cultured by the method of Bianco et al.^10^ Briefly, the biopsies, from the posterior septum close to the cribriform plate of the SD rat, were placed on ice in Dulbecco's modified Eagle's medium/Ham F12 (DF12; JRH), incubated for 45 min at 37 °C in Dispase II (2.4 U/mL in Puck's solution; Boehringer, Mannheim, Germany), after which the lamina propria was separated from the epithelium and washed in Hank's balanced salt solution (HBSS; JRH), then cut into small pieces and incubated in collagenase (0.25%; Type I; Sigma, St. Louis, MO) in DMEM/F12 for 10 min at 37 °C. The tissue was pelleted by centrifugation, dissociated into single cells by trituration, and then resuspended in DF12 supplemented with fetal calf serum (FCS, 10%) and plated into poly-l-lysine coated tissue culture flasks (1 μg/cm^2^; Sigma). Cells were grown in this medium at 37 °C/5% CO_2_ for 48 h, after which the medium was changed to DF12 supplemented with neurotrophin-3 (50 ng/mL; Alamone Labs, Jerusalem, Israel), a process that enhances OEC proliferation.^10^

OB-OECs were cultured by the method of Nash et al.^17^ Briefly, cells from the outer layers of SD rat olfactory bulb were collected in 10 mL of PBS solution. The tissues were mixed with 1% trypsinization, then washed with growth medium (DF12 50:50, Invitrogen Corp, Carlsbad, CA, and 10% FCS, Invitrogen). 20 mL DNAse solution was added to the tissues, and the suspension was triturated for 10 min. The cells were then plated on poly-d-lysine-coated dishes, with fresh growth medium every 4 days.

S100, GFAP, and p75NTR were used as makers to identify OECs.

### Preparation of PLA/chitosan conduit

PLA/chitosan conduits and collagen were prepared with 3.5 mm-long, outer diameter of 1.4 mm and inner diameter of 1.2 mm, to bridge the defect and imitate the ECM of facial nerve.

### Cell transplantation

Cells were scraped from the dish to produce a small, approximately 1 mm-diameter gelatinous bolus that was placed onto the transplant site by use of fine-point needle. 1.0 × 10^7^ cells were required per transplant.

**Figure 1:**
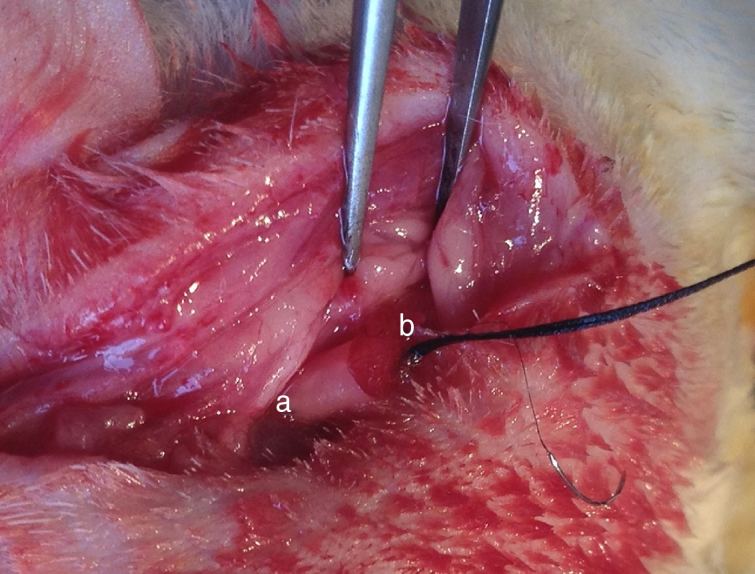
Suture of the injured facial nerve and the conduit (a, conduit; b, the distal end of the facial nerve).

Institutional guidelines regarding animal experimentation were followed (201510129A). 36 SD male rats were randomly divided into three different groups depending on the transplanting cells (12 rats per group): Group A, OM-OECs; Group B, OM-OECs; Group C, DF12/FBS. Rats were anesthetized and submitted to sectioning of the facial nerve near its emergence through the mastoid foramen. The main trunk of the facial nerve was transected, and the two ends detracted to form a 5 mm gap. The gap was immediately bridged using the PLA/chitosan conduit and the ends and conduits were epineural sutured (Fig. 1). A bolus of cultured candidate reparative cells was injected into the collagen sponge in the conduit. In the control group, the proximal part of the extracranial facial nerve was exposed.

After the OECs transplant, the three groups’ rats were divided into two sub-groups (6 rats per group) depending on the post-op time to test: 4 weeks and 8 weeks.

### Evaluation

The rats were scarified for the facial muscle function score, neuro electrophysiological examination, histology analysis, and fluorescent retrograde tracing, respectively, to evaluate the regeneration of facial nerve and to analysis the differences among the three groups.

### Gross observation and facial muscle functional score assessment

Facial muscle function was scored within 0–4. Briefly, eye closure and blinking reflex, vibrissae movement and positioning were observed.

0 – no eye closure, blinking reflex, nor vibrissae movement, with unparalleled vibrissae positioning;

1 – weak eye closure, blinking reflex, and vibrissae movement, without voluntary movement, with unparalleled vibrissae positioning;

2 – weak eye closure, blinking reflex, and vibrissae movement, with voluntary movement, with unparalleled vibrissae positioning;

3 – eye closure, blinking reflex, and vibrissae movement is nearly normal, with nearly paralleled vibrissae positioning;

4 – eye closure and blinking reflex, vibrissae movement is normal, with paralleled vibrissae positioning.

### Neuro electrophysiological examination

Four weeks and 8 weeks post-surgery, 4 rats were chosen randomly from each group to test for facial nerve evoked potentials. The rats were anesthetized and the previous incision was cut to re-expose the PLA/chitosan conduit and facial nerve. The normal facial nerve on the other side was also exposed. The facial nerve evoked potentials were examined using an electrophysiological tester (Keypoint, Frederiksberg, Denmark). A stimulating needle was placed between the proximal end of conduit and normal facial nerve. A recording needle was placed in the orbicularis oris. The stimulating intensity was 2.0 mA, the stimulating frequency was 1 Hz, the sensitivity was 5 ms/D, and the stimulating time was 0.1 ms. Compound muscle action potential was measured and the latency period and peak amplitude were recorded on the normal and regenerative nerve.

### Histological observation

Four weeks and 8 weeks post-surgery, after electrophysiological examination, 4 rats were sacrificed from each group for histology. The regenerative facial nerves with PLA/chitosan conduits were removed and examined.

The total nerve section area was divided into two sampling fields from the distal end to proximal end. One field was chosen randomly for longitudinal section for light microscopy examination, while the other was used for cross section for electron microscopy.

For the light microscopy examination, the nerve sections were cut in 1 μm, fixed in 10% buffered formalin for 24 h, dehydrated, and embedded in paraffin blocks. The sections were de-waxed and stained with hematoxylin and eosin immunohistochemistry. The stained sections were examined at 100× magnification.

For the electron microscopy examination, ultrathin sections (0.1 μm) were obtained. The nerve sections were fixed in glutaraldehyde buffered in cacodylate overnight, washed, and then stored in cacodylate buffer. Samples were fixed in osmium tetroxide, washed in a graded alcohol series, embedded in Epon812, cut of 100 nm in each, and then stained with toluidine blue. The sections were observed on a transmission electron microscope at various magnifications. Five randomly selected fields of view at 3600× magnification were used to calculate the total number, fiber diameter, and myelin sheath thickness.

### Fluorescent retrograde tracing

Four weeks and 8 weeks post-surgery, the left 2 rats from each group were test for fluorescent retrograde tracing. The rats were anesthetized and the previous incision was cut to re-expose the PLA/chitosan conduits and regenerative facial nerve. The normal facial nerve on the other side was also exposed. 2 uL 3% solution of fluorescent gold FG were injected to the distal end of the regenerative facial nerve using micro-syringe. Then all the cuts were sutured. The rats survived 5d after the procedure and then sacrificed. Saline and then 4 °C 0.1 moL PBS with 4% paraformaldehyde and 0.5 mL glutaraldehyde were used for perfusion right atrial appendage through the ascending aorta. The brainstems were collected and fixed with 4% paraformaldehyde for 24 h, 30% sucrose for 48 h, rinsed with water, and embedded with OCT. The brainstems were then cut into serial sections of 25 μm, and examined via post-fluorescence microscope. The number of the golden yellow fluorescent labeled cells with intact nucleus or soma and obvious projections was counted.

### Statistics data analysis

Data were presented as mean ± standard deviation and analyzed by ANOVA (post hoc Tukey test). The level of significance was set to <0.05. GraphPad Prism version 5.0 (GraphPad Software, Inc, San Diego, Calf.) was used.

## Results

OECs from rat OM and OB were successfully isolated and cultured, and highly purified and stable OECs were obtained. All rats survived after the operation.

### Facial muscle function score

**Figure 2:**
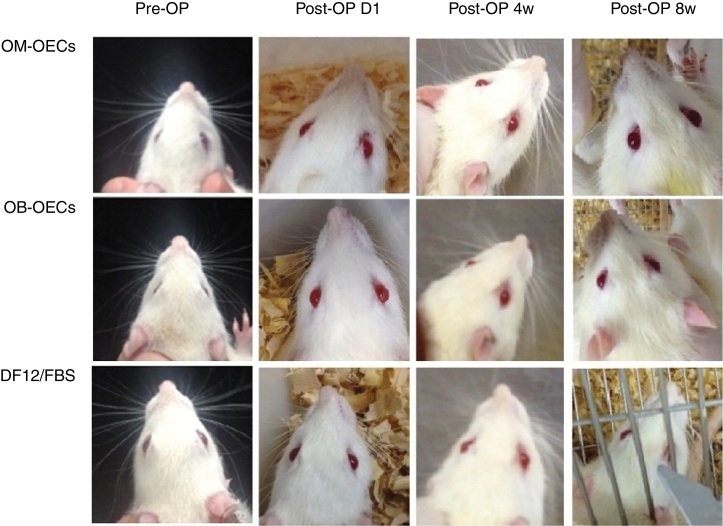
Gross observation of the three groups of rats 4 and 8 weeks after the procedure.

Immediately after a facial nerve lesion, all rats presented complete facial paralysis on the surgery side, with whisker-pad, failure to move the eyelids, sunken beards, and weakened wink reflection. Over the following weeks, facial function improved in all groups, but muscle movement remained worse compared with the healthy side (Fig. 2).

The facial function score in Group A, B and C, after 4 and 8 weeks, were shown in Table 1. After 4 and 8 weeks, there were significant differences in facial nerve function scores between Group A, B and C, and there were no differences between Group A and B (Table 1).

**Table 1:** Facial function score (*x* ± *s*).

Group (*n* = 6)	4 weeks	8 weeks
A: OM-OECs	2.17 ± 0.41	3.00 ± 0.63
B: OB-OECs	2.00 ± 0.63	2.83 ± 0.75
C: DF12/FBS	1.17 ± 0.41	1.83 ± 0.41
Control	4	4
*p*-value (A vs. B)	0.30	0.34
*p*-value (A vs. C)	0.00088	0.0023
*p*-value (B vs. C)	0.01	0.01

### EMG results

In electromyography, all rats experienced the prolongation of time-latency and decrease of voltage amplitude to stimuli after injury. Decrease of time-latency and threshold and increase of amplitude were observed during the recovery.

The latency of facial nerve evoked potentials and the amplitude of facial nerve evoked potentials in Group A, B and C, after 4 weeks and 8 week are shown in Table 2. After 4 weeks, there were significant differences in latency and amplitude between Group A, B and C, and there was no differences between Group A and B. After 8 weeks, there were significant differences in the latency and amplitude among Group A, B and C, latency A < B < C, amplitude A > B > C (Table 2).

**Table 2:** Facial nerve evoked potentials (*x* ± s).

Group (*n* = 4)	Latency period (ms)		Amplitude (mV)	
	4 weeks	8 weeks	4 weeks	8 weeks
A: OM-OECs	2.91 ± 0.04	2.13 ± 0.03	1.90 ± 0.08	2.44 ± 0.03
B: OB-OECs	3.01 ± 0.05	2.56 ± 0.02	1.89 ± 0.08	1.99 ± 0.06
C: DF12/FBS	4.33 ± 0.09	3.87 ± 0.07	0.80 ± 0.03	0.93 ± 0.05
Control	1.89 ± 0.04	1.80 ± 0.14	2.80 ± 0.14	2.86 ± 0.12
*p*-value (A vs. B)	0.011	0.00000077	0.43	0.000048
*p*-value (A vs. C)	0.0000031	0.00000057	0.0000098	0.000000032
*p*-value (B vs. C)	0.0000016	0.0000066	0.00001	0.00000012

### Histological observation

#### Gross pathology

**Figure 3:**
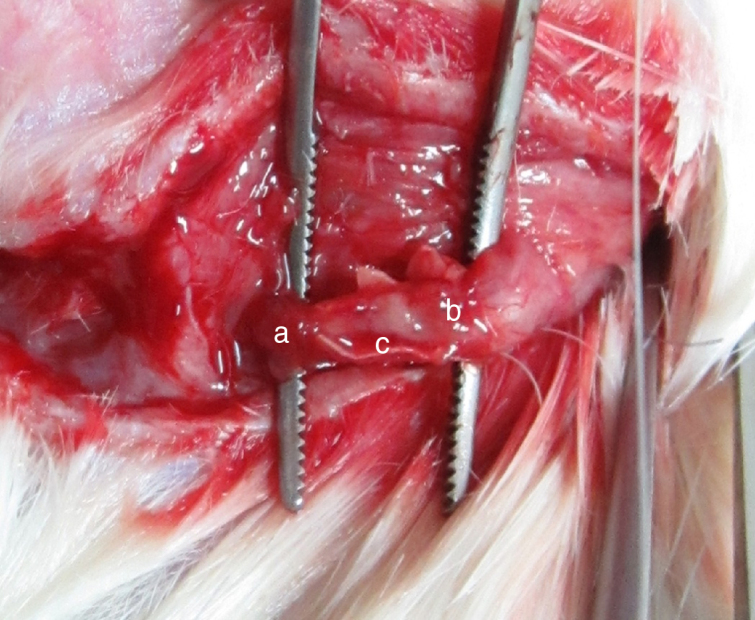
The regenerative facial nerve in the conduit (a, conduit; b, the distal end of the facial nerve; c, regenerative nerve).

Four weeks after the surgery, nerve defects were repaired by regenerative nerve in all groups. The nerves were easy to separate without adhesion and scar formation. There was no neuroma formation in the proximal and distal end of regenerative nerve. PLA/chitosan conduits became thinner and easy to fracture, but still intact. Eight weeks after the surgery, PLA/chitosan conduits were partially absorbed; and the conduit walls were not intact. In Group A and B, the regenerative facial nerve had good continuity, without thinning and hardening. The appearances of the regenerative nerves were similar to normal (Fig. 3).

#### Light microscopic findings

Normal facial nerve possesses the myelinated axons with thick uniform myelin layer, composed of one to five fascicles enveloped by epineuria with spindle shaped cells, and axons were regularly arranged and had similar diameters (original magnification 2000).

Four weeks after surgery, in Group A, myelinated axons with large diameter were nearly normal, but small-diameter myelinated axons were irregular. In Group B, occasional myelinated axon was presented with many Schwann cells with variable morphology. In Group C, there were a large amount of degenerated dark myelin clumps without axons (bungner bands) and severe fibrosis, and the perineurium was a condensation of loose connective tissue.

**Figure 4:**
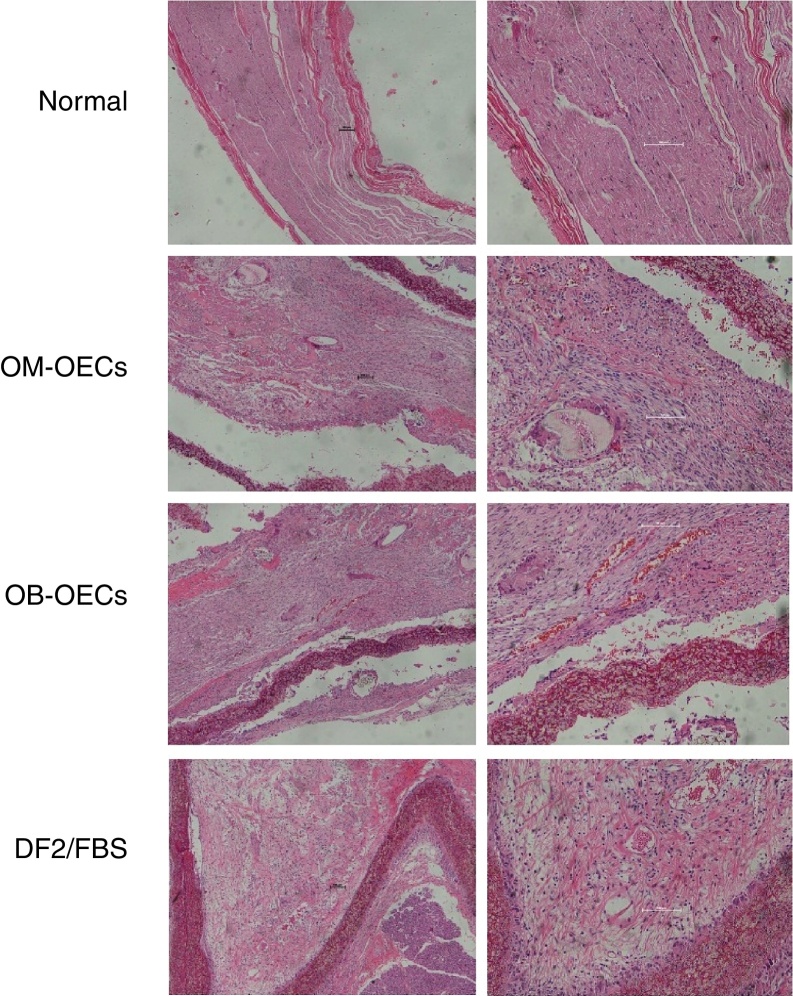
The histological observation of the regenerative nerve in three groups under light microscopy.

Eight weeks after surgery, the maturity of the regeneration nerve was better in all groups compared with that of 4 weeks. In Group A, regenerative nerve bundles were similar to normal nerves, with uniform size. Myelins were dense, with no deterioration. Schwann cells were well distributed, with similarly sized and shaped cell nuclei. In Group B, rich vessels were seen in the epineurium, bundled regenerative nerve fibers, and many neovessels in the nerve fiber bundles. In Group C, there were fewer regenerative axons with uneven distribution and poor development, and a lot of fibrous connective tissue (Fig. 4).

#### Electron microscopy findings

Table 3 shows the number of myelinated nerve fibers, the nerve fiber diameter, and the thickness of the myelin sheath in Group A, B and C, after 4 weeks and 8 weeks respectively.

**Table 3:** Electron microscopy finds (*x* ± *s*).

Group (*n* = 4)	Number of myelinated nerve fibers		Diameter of nerve fibers (μm)		Thickness of myelin sheath (μm)	
	4 weeks	8 weeks	4 weeks	8 weeks	4 weeks	8 weeks
A: OM-OECs	1911.50 ± 78.10	2743.75 ± 82.47	3.33 ± 0.15	4.73 ± 0.12	0.71 ± 0.06	0.89 ± 0.06
B: OB-OECs	1899.75 ± 60.46	2319.50 ± 91.83	3.21 ± 0.16	3.96 ± 0.12	0.69 ± 0.05	0.75 ± 0.02
C: DF12/FBS	1763.50 ± 83.33	2008.50 ± 134.10	2.39 ± 0.06	3.10 ± 0.03	0.40 ± 0.04	0.55 ± 0.02
Control	3022.50 ± 133.75	3096.75 ± 110.91	5.65 ± 0.36	5.99 ± 0.36	0.93 ± 0.04	0.94 ± 0.06
*p*-value (A vs. B)	0.41	0.00025	0.16	0.00005	0.31	0.0070
*p*-value (A vs. C)	0.021	0.00012	0.00017	0.000025	0.00014	0.00034
*p*-value (B vs. C)	0.021	0.0055	0.00041	0.00021	0.000066	0.0000039

**Figure 5:**
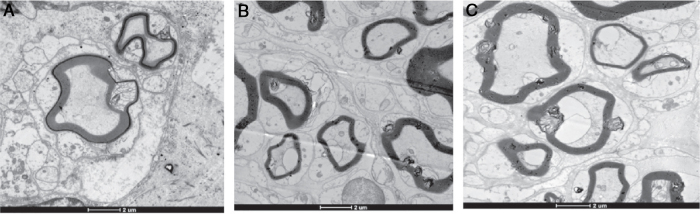
The histological observation of the normal facial nerve the regenerative nerve in three groups under electron microscopy.

After 4 weeks, the number of regenerative nerve fibers, the diameter of the fiber, and the thickness of myelin sheath in Group A and B are superior to that of Group C, and there were no differences between Group A and B. After 8 weeks, the number of regenerative nerve fibers, the diameter of the fiber, and the thickness of myelin sheath in Group A are superior to that of Group B, and that of Group B are superior to that of Group C (Fig. 5).

#### Fluorescent retrograde tracing

Table 4 shows the average numbers of FG-labeled cells in the facial nucleus at 4 and 8 weeks after cell transplantation in Group A, B and C, respectively.

**Table 4:** number of FG-labeled cells (*x* ± *s*).

Group (*n* = 2)	4 weeks	8 weeks
A: OM-OECs	175.00 ± 18.38	237.50 ± 12.02
B: OB-OECs	154.00 ± 5.66	191.00 ± 8.49
C: DF12/FBS	53.50 ± 6.36	96.00 ± 9.90
Control	271.50 ± 20.51	279.50 ± 12.02
*p*-value (A vs. B)	0.17	0.028
*p*-value (A vs. C)	0.0023	0.0034
*p*-value (B vs. C)	0.0019	0.0050

**Figure 6:**
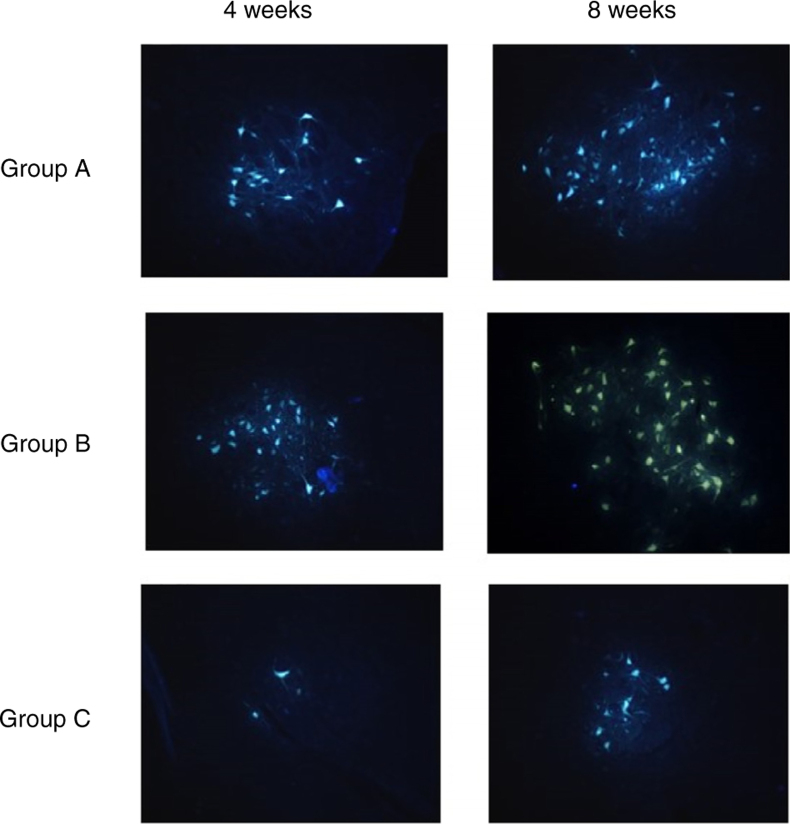
The FG-labeled cells in the facial nucleus of the three groups of rats 4 and 8 weeks after the procedure.

After 4 weeks, there were significant differences in FG-labeled cell number between Group A, B and C, and there was no differences between Group A and B. After 8 weeks, there were significant differences in FG-labeled cell number between Group A, B and C; A > B > C (*p* < 0.01) (Table 4) (Fig. 6).

## Discussion

Glial cells transplantation has been frequently used to promote axonal regeneration in Central and Peripheral Nervous System (CNS PNS). Myelin-forming cells usually used for transplantation are Schwann cells, olfactory ensheathing cells, etc.^18^ OECs are unique glial cells that share some properties with astrocytes and Schwann cells but have some intrinsic properties. Studies showed that OECs could mediate neurotrophic and neuroprotective effects by secretion of chemokines, enhance axonal regeneration and produce myelin after transplantation.^4^ It has been observed that OECs can promote axonal regeneration of CNS and PNS neurons in culture and in vivo,^6^ which make them ideal candidates for cellular transplantation.

Olfactory Bulb (OB) is the most commonly used source to obtain OECs in most studies. However, it is not a clinically favored site for sourcing autologous OECs because of the morbidity associated with surgical access and destruction of the olfactory bulbs to obtain the cells. OECs can also be isolated from the olfactory mucosa (OM). Previous studies showed that OM-OECs also have the capability to promote nerve regeneration,^13^ but there are very few studies about the differences between OM-OECs and OB-OECs on their effect of nerve regeneration.

It is known that peripheral nervous system has the ability to achieve functional recovery after nerve injury, and rat facial neurons are able to bridge gaps of up to 10 mm. There are some studies about different methods to facilitate facial nerve regeneration,^19^, ^20^, ^21^, ^22^, ^23^, ^24^ and some studied OECs transplantation in facial nerve injury,^23^, ^24^ but most of the studies focused on the collateral axonal branching of the regenerative facial nerve. Recently there is a study about human olfactory stem cells from OM could improve the recovery of rat facial nerve injury in two measurements: whisker movement and electroneuronography analyses.^24^ In our study, we try to use different measurements to better evaluate the nerve regeneration outcome of different oriented OECs transplantation after PNS injury.

Facial muscle function is the most objective result of nerve regeneration. The recovery of facial muscle function is the last step of nerve regeneration, which means the regenerative nerve has the capability to control the target muscle. After 4 and 8 weeks, there were significant differences in facial nerve function scores between Group A, B and C, and there was no differences between Group A and B. The result means that facial muscle functions were similar in Group A and B, and among the three groups, the rats in Group C had the worst facial function. It could be concluded from the results that both of OM-OECs and OB-OECs can stimulate the regeneration of injured facial nerve.

Facial nerve evoked potential is the most effective index to show the regeneration degree of the injured nerve. The improvement of the evoked potentials means that the regenerative nerve was able to conduct action potentials to the target tissue. After 4 weeks, there were significant differences in latency and amplitude between Group A, B and C, and there was no difference between Group A and B. After 8 weeks, latency A < B < C, amplitude A > B > C. Studies showed that facial nerve evoked potential is the first sign to show change after effective nerve regeneration, while the muscle function is the last to show the change,^25^ which explains that in our study, after 8 weeks, there were significant differences in the latency and amplitude among Group A, B and C, but there was no differences between Group A and B in facial muscle function scores.

Histological observation gives us a direct observation of the nerve regeneration. Light microscopic findings shows that the regeneration in Group A was better than Group B, and Group B was better than Group C. After 8 weeks, the regenerative nerves in Group A were similar as the normal ones. We calculated the values of the number of the nerve fibers, the fiber diameter of the myelin sheath, and the sickness of myelin sheath in three groups under electron microscopy. The results were in accordance with the findings under light microscopy. These histological observations revealed that, the OECs transplantation can lead to nerve tissue regeneration with large numbers of myelinated nerve fibers, crude fibers, and larger myelin thickness and volume in the transplanted graft, and OM-OECs transplantation seemed to have better results in long term.

Fluorescent retrograde tracing results showed that whether the regenerative nerves had set up the efficient connection to the facial nucleus. The histological structure of the regenerative nerve connecting two ends of injured nerve is only the first step for complete functional recovery. It does not mean the regenerative nerve has achieved complete functional restoration. Only the regenerative nerve has efficient connection with the facial nucleus, means the whole nerve pass is set up.^26^ In this study we counted the FG-labeled cells in the facial nucleus at 4 and 8 weeks after cell transplantation. The results revealed that OECs transplantation stimulated the connection between regenerative nerve and facial nucleus, and the transplantation of OM-OECs had a better effect of nerve connection compared with OB-OECs in the long term.

In this study, the facial nerve regeneration was evaluated in four different ways: functional assessment of the terminal organ, electrophysiology, histology (qualitative and quantitative), assessment of whole nerve pass way connection. Almost all the four evaluations had similar results: OECs could promote the regeneration of injured facial nerve. Compared with OB-OECs, OM-OECs were more effective in promoting nerve regeneration, and this difference was more significant 8 weeks after the transplantation than 4 weeks.

These results showed that OECs transplantation with nerve conduits may acts as a powerful tool to enforce peripheral nerve regeneration under adverse conditions, e.g., long deficit between the two ends, and OM-OECs could be a more convenient and effective source for transplantation. There are still remaining questions that need to be addressed further. For instance, postparalytic syndrome (abnormally associated movements after facial nerve transaction) is caused by a given muscle group reinnervated by misrouted axonal branches. Olfactory ensheathing cells have been shown to reduce axonal sprouting and stimulate axonal regeneration after transplantation into the spinal cord. Could OECs transplantation also reduce sprouting of a damaged peripheral pure motor nerve? Is OECs transplantation more effective compared with simple facial-facial anastomosis? What is the mechanism of the transplanted OECs promoting nerve regeneration?

## Conclusion

We discovered that OECs with nerve conduit could improve the recovery of injured facial nerve, and OM-OECs are more effective for facial nerve regeneration compared with OB-OECs 8 weeks after the transplantation. These results could cast new light on finding new treatment for facial nerve defect, and furnish the foundation of auto-transplantation of olfactory mucosa OECs in periphery nerve injury. Larger studies are needed to confirm our findings and unravel the underlying mechanisms.

## Funding

This work was supported by National Natural Science Foundation of China (no. 81300837).

## Conflicts of interest

The authors declare no conflicts of interest.
